# Performance Evaluation of IEEE 802.11ah Networks With High-Throughput Bidirectional Traffic

**DOI:** 10.3390/s18020325

**Published:** 2018-01-23

**Authors:** Amina Šljivo, Dwight Kerkhove, Le Tian, Jeroen Famaey, Adrian Munteanu, Ingrid Moerman, Jeroen Hoebeke, Eli De Poorter

**Affiliations:** 1 Department of Information Technology, Ghent University-imec, IDLab, Technologiepark-Zwijnaarde 15, B-9052 Ghent, Belgium; dwight.kerkhove@ugent.be (D.K.); ingrid.moerman@ugent.be (I.M.); jeroen.hoebeke@ugent.be (J.H.); eli.depoorter@ugent.be (E.D.P.); 2 Department of Mathematics and Computer Science, University of Antwerp-imec, IDLab, Middelheimlaan 1, 2020 Antwerpen, Belgium; le.tian@uantwerpen.be (L.T.); jeroen.famaey@uantwerpen.be (J.F.); 3 Department of Electronics and Informatics (ETRO), Vrije Universiteit Brussel, Pleinlaan 2, B-1050 Brussels, Belgium; acmuntea@etrovub.be

**Keywords:** IEEE 802.11ah, Internet of Things, TCP/IP, bidirectional traffic, scalability, streaming, Traffic Indication Map (TIM), Restricted Access Window (RAW)

## Abstract

So far, existing sub-GHz wireless communication technologies focused on low-bandwidth, long-range communication with large numbers of constrained devices. Although these characteristics are fine for many Internet of Things (IoT) applications, more demanding application requirements could not be met and legacy Internet technologies such as Transmission Control Protocol/Internet Protocol (TCP/IP) could not be used. This has changed with the advent of the new IEEE 802.11ah Wi-Fi standard, which is much more suitable for reliable bidirectional communication and high-throughput applications over a wide area (up to 1 km). The standard offers great possibilities for network performance optimization through a number of physical- and link-layer configurable features. However, given that the optimal configuration parameters depend on traffic patterns, the standard does not dictate how to determine them. Such a large number of configuration options can lead to sub-optimal or even incorrect configurations. Therefore, we investigated how two key mechanisms, Restricted Access Window (RAW) grouping and Traffic Indication Map (TIM) segmentation, influence scalability, throughput, latency and energy efficiency in the presence of bidirectional TCP/IP traffic. We considered both high-throughput video streaming traffic and large-scale reliable sensing traffic and investigated TCP behavior in both scenarios when the link layer introduces long delays. This article presents the relations between attainable throughput per station and attainable number of stations, as well as the influence of RAW, TIM and TCP parameters on both. We found that up to 20 continuously streaming IP-cameras can be reliably connected via IEEE 802.11ah with a maximum average data rate of 160 kbps, whereas 10 IP-cameras can achieve average data rates of up to 255 kbps over 200 m. Up to 6960 stations transmitting every 60 s can be connected over 1 km with no lost packets. The presented results enable the fine tuning of RAW and TIM parameters for throughput-demanding reliable applications (i.e., video streaming, firmware updates) on one hand, and very dense low-throughput reliable networks with bidirectional traffic on the other hand.

## 1. Introduction

The Internet of Things (IoT) is advancing towards 26 billion connected units by 2020 according to estimations [1]. For this to become a reality, it is necessary to provide connectivity for a large number of energy-constrained smart devices in an energy efficient manner, while maximizing throughput and range. As endpoints in IoT are typically embedded devices, often battery powered and deployed in outdoor environments, they require scalable low-power wireless technologies. Existing low-power wireless technologies used in IoT can be divided in two classes: (1) Wireless Personal Area Networks (WPAN) and (2) Low-Power Wide Area Networks (LPWAN). WPAN technologies such as Radio-Frequency Identification (RFID), ZigBee, and Bluetooth , although applicable to low-power device communication, have a number of constraints that limit their capabilities, mostly regarding the maximum number of connected devices, throughput and transmission range. On the other hand, LPWAN technologies such as LoRa and SigFox provide long range communication (up to 15 and 50 km, respectively), but have a very low throughput (up to 50 and 0.1 kbps, respectively).

A new wireless standard, IEEE 802.11ah [2] (also known as Wi-Fi HaLow), introduces a trade-off between throughput and range and fills the gap between WPAN and LPWAN. IEEE 802.11ah operates in unlicensed sub-GHz frequency bands (863–868 MHz in Europe, 755–787 MHz in China and 902–928 MHz in North-America) and can provide connectivity between at most 8192 low-power devices at rates from 150 kbps to 78 Mbps over a range up to 1 km. Hence, IEEE 802.11ah supports much higher throughput than existing LPWAN technologies and both higher throughput and range than WPAN technologies. Moreover, LoRa and SigFox have severe restrictions concerning downlink (DL) traffic. LoRaWAN class A devices can only receive a downlink packet in response to an uplink (UL) packet at the same (for RX1DROffset=0) or lower (for other values of RX1DROffset and RX2) data rate than the uplink [3]. SigFox allows only 140 uplink messages (up to 12 bytes) and four downlink messages (up to 8 bytes) per day, which makes reliability infeasible as only a few uplink packets can even be confirmed. Those restrictions make high-throughput combined uplink and downlink (UL/DL) communication infeasible for both technologies. Ergo, IEEE 802.11ah fills the gap when it comes to reliable and high-throughput applications over a somewhat smaller area. It offers the same data rate for downlink and uplink traffic and, hence, enables applications with frequent actuation (i.e., control-loops), high-throughput applications (e.g., video streaming, firmware updates) and reliable monitoring (i.e., acknowledging every transmitted packet). So far, a number of IEEE 802.11ah performance improving algorithms have been proposed, but only for typical Wireless Sensor Network (WSN) monitoring traffic. Monitoring in WSNs corresponds to periodically transmitting the measured values and sleeping for the remainder of the interval. Almost 80% of available research on Wi-Fi HaLow data-link layer only addresses use cases with uplink traffic. Only some research addresses specific use cases with either downlink or bidirectional traffic (energy efficiency, latency, hidden node mitigation).

Next to this, IEEE 802.11ah introduced the Restricted Access Window (RAW) feature for Media Access Control (MAC). However, it does not define how to configure Traffic Indication Map (TIM) and RAW mechanism. This freedom can lead to significant improvements in performance, but also to degradation of the network, as the performance of these mechanisms is greatly dependent on traffic patterns. To make the most of both TIM and RAW and exploit their benefits, it is necessary to configure them properly. Today, there is a lack of practical knowledge on both (1) its operation with Resource Allocation (RA), which can be used for downlink scheduling, and (2) its performance in parallel with TIM segmentation. Available RAW (re)configuration algorithms are only applicable to WSN scenarios given that they are based on uplink throughput and/or energy optimization. Moreover, MAC mechanisms for downlink scheduling have been barely investigated, let alone optimized.

Therefore, we investigated the combined performance of RAW and TIM with UL/DL traffic patterns. We varied density, Modulation and Coding Schemes (MCSs) and configurations of RAW, TIM and Transmission Control Protocol (TCP) through more than 40,000 simulations in the ns-3 network simulator in two scenarios: (1) Video streaming that needs reliable and high-throughput (Section 4.2) and (2) Reliable monitoring scenario (Section 4.3) where each packet is acknowledged at the application layer. The first scenario implies a set of motion cameras that stream if they detect motion, whereas the second implies a very dense network of reliable sensors. Reliability in both scenarios is achieved through the use of TCP. We shortened the Round Trip Time (RTT) by introducing the *immediate reply* mechanism. We also demonstrated that IEEE 802.11ah can support legacy Internet traffic by way of verbose TCP that is rarely suitable for IoT applications. TCP acknowledgements represent DL traffic in both scenarios. In the first scenario, 92% of the total traffic in simulated networks was UL, whereas the rest (8%) was DL. In the second scenario, 67% of the total traffic was UL and 33% was DL.

By enabling the described experiments and assessing the results, this article makes the following key contributions:

- Detailed analysis of the influence of RAW and TIM grouping (in parallel) on attainable scalability, throughput, latency and energy-efficiency considering bidirectional communication,
- Insights in the trade-off between scalability and throughput, along with configuration best-practices for achieving the desired performance,
- An immediate reply mechanism at the Access Point to reduce downlink latency,
- An extension of the ns-3 IEEE 802.11ah module [4], namely implementation of: (1) Delivery TIM (DTIM), (2) TIM segmentation, (3) non-Cross-Slot Boundary (non-CSB), (4) transmission scheduling for DL frames and (4) immediate reply mechanism.

The remainder of this article is structured as follows. Section 2 introduces the related research on IEEE 802.11ah. Section 3 gives an overview of the 802.11ah MAC features relevant for this paper. Furthermore, Section 4 presents performed experiments, namely a video streaming scenario (Section 4.2) and a reliable monitoring scenario (Section 4.3). Setups of the two experiments and obtained results are discussed in the corresponding sections. Section 4 also presents the simulation environment (Section 4.1), theoretical limits (Section 4.4) and a discussion on delay tolerance of TCP (Section 4.5). Finally, conclusions are summarized in Section 5.

## 2. Related Work

Even before the first draft of IEEE 802.11ah appeared in October 2013, the research community had already demonstrated its interest in its performance. That interest led to early evaluations and overviews of the key features of the technology, describing the advantages and challenges in the physical and MAC layer design [5,6,7,8,9,10]. Furthermore, evaluations of key features led to novel solutions for performance improvement of the technology, namely improvements in energy efficiency, throughput, latency and hidden node mitigation, especially on the MAC layer. Available research regarding optimization mechanisms on the MAC layer are summarized in Table 1.

Being the most popular MAC layer feature according to the number of published papers, the RAW feature is utilized in most MAC layer algorithms that improve network performance in some way. However, even though RAW can improve scalability in highly dense deployments, its performance depends on current network conditions. Most of the available algorithms and evaluations consider uplink traffic only. In that manner, Charania et al. [17] developed the Delay and Energy Aware RAW Formation (DEARF) scheme to support delay sensitive devices along with other delay tolerant devices. They eliminated contention for transmission by employing Resource Allocation feature of RAW for delay sensitive stations. However, only uplink traffic is considered for both delay sensitive and insensitive stations. Regarding throughput optimization, Tian et al. [19,20] developed a real-time Traffic-Adaptive RAW Optimization Algorithm (TAROA) that improves uplink throughput in dynamic and heterogeneous WSNs. Nawaz et al. [18] argue that the duration of each RAW slot should be chosen according to the size of the group, which has proven to increase uplink throughput. Chang et al. [25] proposed a load-balanced sensor grouping for RAW based on set partitioning that assumes that the Access Point (AP) knows the (static) traffic demand of each station. Qutab-Ud-Din et al. [28] analyzed RAW performance in the non-Cross Slot Boundary use case under various holding schemes. They also proposed new holding schemes for RAW based on back-off states of the stations. Khorov et al. [27] developed a mathematical model that allows for finding (1) the distribution of time required for an arbitrarily chosen station to transmit its frame, and (2) the distribution of time required for all stations to transmit their frames in a RAW slot. They assumed that the AP does not transmit anything except acknowledgements (ACKs), there are no hidden nodes and transmission errors can only be caused by collisions. This model enables calculating the minimal RAW slot duration required for (1) an arbitrary chosen station to successfully transmit its frame with some predefined probability, and (2) all stations to successfully transmit their frames with some predefined probability; thus, it is only valid for WSN scenarios.

One could expect downlink traffic in hidden node mitigation studies. However, only Damayanti et al. [16] assume the network is saturated with downlink traffic (limiting transmission to a device once per RAW) and propose RAW grouping based on a previously constructed carrier-sensitivity table, whereas Yoon et al. [23] identify hidden nodes by non-acknowledged Power-Saving-Poll (PS-Poll) transmissions (i.e., uplink). Dong et al. [24] also assume that the network is saturated with uplink traffic and groups the stations according to their geographical position.

An actuation scenario is investigated by Badihi et al. [15] who analyzed performance of IEEE 802.11ah in a connected lightning use case considering latency and power consumption at the actuator. They investigated how MCS, TIM segmentation and DTIM period influence DL latency and energy consumption and concluded that 2 MHz channel bandwidth leads to lower DL latency compared to 1 MHz, that there is a trade-off between latency and power consumption in terms of DTIM period (i.e., short DTIM period contributes to lower latency, but increases power consumption) and that using broadcast/multicast for groups of actuators lowers the latency compared to serving the same group with unicast transmissions. Bankov et al. [13] proposed a solution for choosing a suboptimal Protected Interval (PI) duration, where PI represents a time interval within a beacon interval reserved for Power-Saving (PS) stations, whereas the rest of the beacon interval represents the Shared Interval in which all stations (both PS and active stations) can transmit their data. PI is merely an abstraction of time-restricting mechanisms such as RAW. They consider heterogeneous UL/DL traffic; however, they validated their model with a small number of stations without TIM segmentation.

Kim and Chang [12] and Bel et al. [14] optimized the energy consumption in IEEE 802.11ah networks that support bidirectional traffic patterns. Bel et al. [14] proposed a novel channel access protocol for IEEE 802.11ah based on a smart use of Channel Access Slots, in which the RAWs can be divided. However, this study is based on an outdated draft of the IEEE 802.11ah standard. The standard published in December 2016 defined RAW in a somewhat different way. Kim and Chang [12] investigated the TIM and page segmentation scheme and proposed a method that (selectively and dynamically) temporarily changes the TIM group membership of nodes and rearranges their traffic to maximize overall sleeping intervals without causing delay to data delivery. Finally, Kureev et al. [11] studied the energy efficiency of 802.11ah in a heterogeneous Wi-Fi network and proposed a simple and fairly accurate mathematical model to calculate the average throughput and energy consumption of a 802.11ah network, considering both TIM segmentation and RAW. Their model enables choosing the number of TIM groups that provides close to optimal energy consumption of PS stations and throughput of active stations.

This paper presents the interplay between TIM segmentation and RAW grouping in demanding scenarios such as high-throughput or high-density considering reliable bidirectional traffic. It shows limitations and interconnections between the two features and suggests how to combine them in order to achieve the desired trade-off between scalability and throughput on one hand, and latency and energy consumption on the other hand, for bidirectional traffic patterns. As such, we aim to fill the existing gap in research on general network performance (considering throughput, reliability, latency and energy) with bidirectional traffic patterns by utilizing the two most popular MAC features of IEEE 802.11ah, RAW and TIM, working in parallel. Furthermore, we demonstrate how IEEE 802.11ah can be used in IoT to support legacy Internet traffic and discuss the issues of using TCP over IEEE 802.11ah. This performance evaluation enables nearly optimal TIM and RAW configuration in compliance with network demands. We hope this work can contribute to improved interoperability possibilities between TIM and RAW and perhaps inspires the design of novel standard-compliant adaptive optimization algorithm(s) that would be applicable to use cases with heterogeneous traffic patterns.

## 3. IEEE 802.11ah Overview

**Figure 1:**
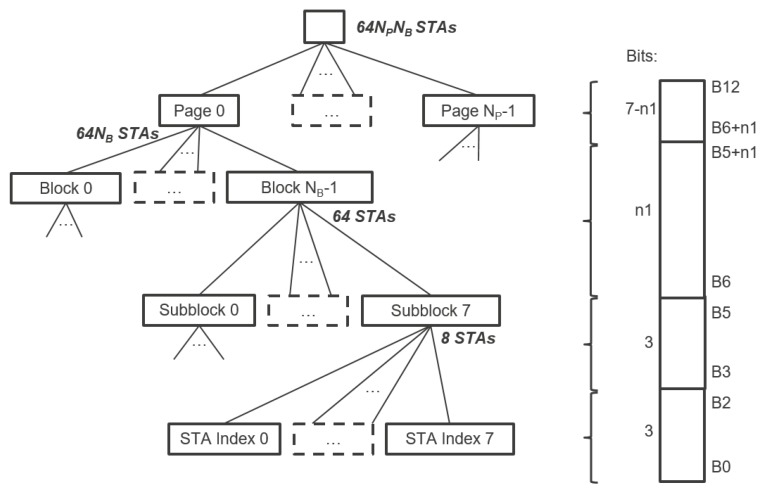
TIM segmentation distributes the stations in a hierarchy, enabling effective management of a large number of stations as well, energy conservation and contention reduction in a structured manner. Each station is assigned a unique AID that represents the station in a hierarchical structure.

Thanks to the novel hierarchical Association Identification (AID) structure shown in Figure 1, IEEE 802.11ah supports up to 8191 associated stations, a much larger value than in previous 802.11 releases. However, this number is a direct consequence of the AID field size (13 bits enabling $2^{13}$ or 8192 unique AIDs, with AID 0 being reserved) and it is yet to be experimentally verified under which circumstances it is possible to connect 8191 devices to one AP in reality. Unlike previous IEEE 802.11 MAC mechanisms, IEEE 802.11ah stations can be configured to use only specific time slices between beacons where they are allowed to contend for the medium and communicate with the AP. This enables the stations to sleep most of the time and only wake up at the beginning of these intervals. The collision probability is greatly reduced by distributing the slices evenly between associated stations.

IEEE 802.11ah defines two modes of operation: (1) TIM mode and (2) non-TIM mode. Stations operating in one of these two modes are referred to as TIM- and non-TIM stations, respectively. TIM stations have periodic access to the medium and are typically used for high bandwidth requirements and receive both down- and uplink access, therefore being the mode used in this research. TIM stations wake up periodically to receive the beacon broadcasted by the AP. On the other hand, in non-TIM mode (power saving mode) stations do not need to periodically wake up to receive beacons. Instead, they transmit at least one PS-Poll or trigger a frame transmission to the associated AP every listen interval. Non-TIM stations are destined to exchange low amounts of data and as such can request buffered DL traffic from the AP or transmit UL traffic any time they wake up.

In order to reduce collisions and interference, the standard introduces the Restricted Access Window (RAW) mechanism (Section 3.2), which enables reserving a specific time window for specific stations only. As such, RAW can be used for restricting access to any specified group of stations. It is useful for improving performance in dense IoT networks where a large number of stations are contending simultaneously [33].

This section describes the TIM and RAW mechanisms in more detail in Section 3.1 and Section 3.2, respectively. TIM and RAW can be used independently or in parallel. Moreover, IEEE 802.11ah has introduced a number of novel MAC mechanisms in comparison to previous Wi-Fi amendments. For more details on these MAC mechanisms, which are summed up in Table 2, we refer the readers to [10], where the authors present a brief overview of all of them, or to the standard itself [2].

### 3.1. Traffic Indication Map (TIM) Segmentation

**Figure 2:**
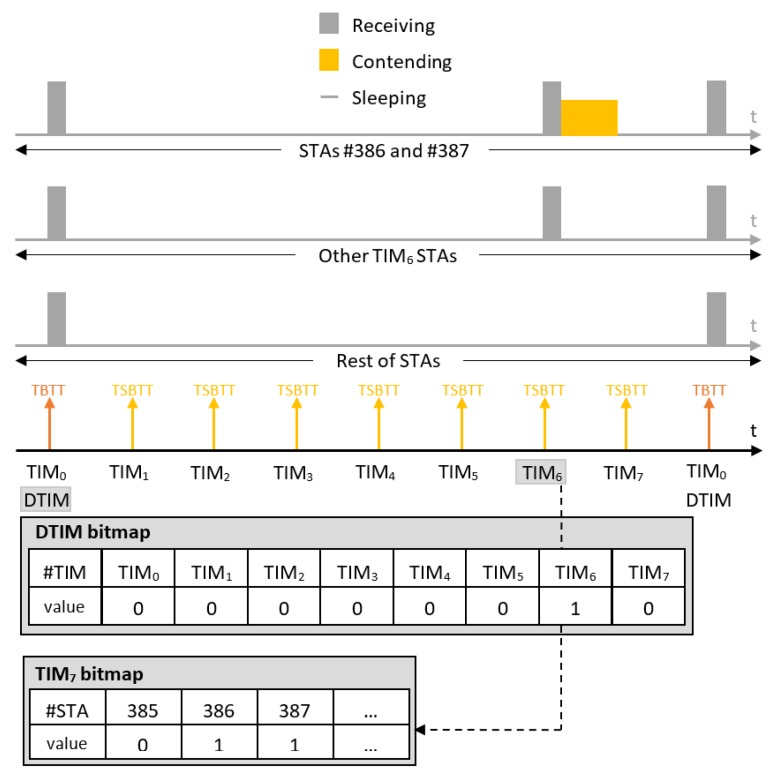
An example of TIM segmentation: all stations wake up to receive the Delivery TIM (DTIM) beacon, only paged TIM groups wake up to receive their TIM beacon and only paged stations within paged TIM groups contend for the medium. In the example, 512 stations are organized in 1 page ($N_{P}=1$) and 8 TIM groups ($N_{B}=8$), where each TIM group has 64 stations.

IEEE 802.11ah introduces TIM segmentation, which distributes the stations in a hierarchy, enabling effective management of a large number of stations as well as energy conservation and contention reduction in a structured manner. Each station is assigned a unique 13-bits AID in the range 1–8191 (AID 0 is reserved for group addressed traffic). The AID represents the station in a hierarchical structure as Figure 1 illustrates. The TIM is structured in a three-level hierarchy: Page ID, Block (TIM Group) and Sub-Block (contains station (STA) indexes). This hierarchy enables the AP to indicate in the bitmap whether a station has pending DL data buffered at the AP on multiple levels. For instance, the AP can indicate that there is DL data pending for TIM group *g* in the DTIM beacon. All stations will check their own TIM group based on their assigned AID and only stations that belong to TIM group *g* will wake up in time for TIM beacon of TIM group *g* to hear which station-index has data to expect, whereas all the remaining stations from other TIM groups can resume sleeping until the next AP announcement. This enables longer sleep periods for stations, which conserves energy and reduces contention as illustrated with an example in Figure 2. Moreover, the hierarchy enables TIM segmentation, which reduces the data length of beacons containing TIMs. In highly dense networks, the TIM would be very long (1 bit per station) without such segmentation. All stations would need to listen to the beacon and receive a very long TIM, including the stations that do not have pending DL data at all. Waste of energy is evident in this case. TIM segmentation does not only reduce energy consumption; it also enables connecting up to 8191 stations thanks to the hierarchical organization of stations into groups.

The traffic-indication virtual bitmap consists of $64N_{P}N_{B}$ bits and is organized into $N_{P}$ pages where each page consists of $N_{B}$ blocks, each block consists of eight subblocks, and each subblock consists of eight bits, as illustrated in Figure 1. The 8-bit *bitmap control field* defines the TIM segmentation. Five bits of this field are reserved for the *Page Slice Number* $N_{B}$, while two bits are reserved for the *Page Index* $N_{P}$. Ergo, $N_{P}$ is at most 4, whereas $N_{B}$ is at most 32, which implies a maximum of 2048 stations in each page and a maximum of 8192 stations overall. As shown in Figure 1, the arbitrary bit number *N* in the traffic-indication virtual bitmap corresponds to the bit number N[0:2] of the N[3:5]-th subblock of the $N[6:5+n_{1}]$-th block of the $N[6+n_{1}:12]$-th page, where $2^{n_{1}}=N_{B}$, N[0] is the least significant bit and N[a:b] represents bits *a* to *b* of the bit number *N*, both inclusive.

When TIM segmentation is used, all TIM-stations wake up at the Target Beacon Transmission Time (TBTT) to receive the beacon carrying the Delivery Traffic Indication Map (DTIM) that indicates which TIM group (block) has buffered DL data at the AP and needs to wake up to receive it. Further, one TIM beacon is broadcast for each TIM group at every Target Short Beacon Transmission Time (TSBTT). The short beacon interval $T_{TIM}$ is announced in the DTIM beacon or during association and represents the number of IEEE 802.11 Time Units (TUs) of 1024 μs between two subsequent TSBTTs. It is an integer in the range 1–65,535. The beacon interval $T_{DTIM}=N_{P}N_{B}\cdot T_{TIM}$ is the number of TUs between two consecutive TBTTs . Apart from the bitmap indicating the stations that have pending DL data buffered at the AP, TIM beacons also announce the TBTT of the next DTIM beacon. Based on the above timings, time intervals between sequential TIM beacons can be in the range 1.024 ms to 67.10784 s and between DTIM beacons in the range of 1.024 ms to 35.790848 min (for a single page). The TIM beacon for the first TIM group is broadcast together with the DTIM beacon.

The term *paged* in this paper refers to the case that there is at least one data packet buffered at the AP. A station is paged (by TIM) if an AP has pending DL traffic for that station, and, in this case, the TIM group to which the STA belongs has to be paged (by DTIM) as well. For example, if the DTIM pages TIM groups $g_{x}$ and $g_{y}$, then all stations assigned to the TIM groups $g_{x}$ and $g_{y}$ will have to wake up at their corresponding TSBTT to receive TIM beacons of their groups. All other stations sleep until the next DTIM beacon, unless they have data to transmit. After receiving TIM beacons of their groups, stations assigned to TIM groups $g_{x}$ and $g_{y}$ will check in the TIM whether they are paged or not. The ones that are paged will contend for the medium, and the rest will go back to sleep until the next DTIM beacon scheduled at TBTT as illustrated in example in Figure 2.

TIM segmentation and its possibilities for energy conservation and latency management have been poorly investigated so far. Kim and Chang [12] proposed a method that (selectively and dynamically) temporarily changes the TIM group membership of nodes and rearranges their traffic in order to reduce the number of unnecessary wake-ups of stations that are not paged in their TIM, but must wake up for receiving the TIM beacon because their TIM group was paged by DTIM. Their method aims to group all paged stations together in order to reduce energy consumption for stations with no DL data in that DTIM interval, without causing additional delays to data delivery. However, their method does not improve DL latency. Charania et al. [17] proposed a delay and energy aware RAW formation scheme where they used TIM segmentation. However, the scheme is only able to broadcast the RAW Parameter Set (RPS) that defines the RAW configuration along with TIM beacon in a single DTIM. They deviated from the TIM specification of the IEEE 802.11ah standard by using short beacon intervals of variable length (short beacon intervals are announced in DTIM beacons and cannot change between two consecutive DTIM beacons, i.e., in TIM beacons). Furthermore, they only consider UL latency where TIM does not play any significant role, whereas delays in bidirectional communication in IEEE 802.11ah (namely, RTT optimization) are a completely different problem. So far, only two research papers show results in relation with TIM segmentation. However, TIM segmentation has much more potential to be exploited, requiring additional research.

### 3.2. Restricted Access Window (RAW)

**Figure 3:**
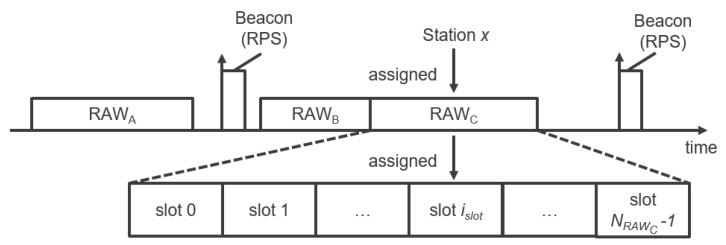
RAW feature: the beacon carries an RPS element that defines the duration of RAW, the number of RAW groups within the beacon interval, their duration, the number of equal-sized slots within each group and the assigned stations.

In order to reduce collisions and interference between stations, the standard introduces the RAW mechanism that could be seen as a combination of deterministic and stochastic media access control mechanisms. RAW enables reserving specific slotted time windows only for specific stations assigned to those windows. During those windows, assigned stations use Enhanced Distributed Channel Access/Distributed Coordination Function (EDCA/DCF) to access the medium within their corresponding slot (Figure 3). RAW can be used for restricting channel access to any specified group of stations. It is useful for achieving performance improvements in dense IoT networks where a large number of stations are contending simultaneously [33]. This section presents the RAW operation.

An AP may allocate one or more RAWs for a group(s) of stations within a (short) beacon interval and broadcast this information using the RAW Parameter Set (RPS) information element carried in the preceding beacon. The RPS element specifies which stations are assigned to RAW(s), the configuration of RAW(s), as well as start time of RAW(s) relative to either the beacon ending time (for the first RAW) or the previous RAW. Each RAW can be divided into time slots referred to as RAW slots, and stations assigned to RAW are evenly split across the RAW slots using round robin assignment. If a station belongs to a RAW group, it is allowed to contend for medium access at the start of its assigned RAW slot and will not contend for medium access within any other RAW slot during that RAW. The number of slots $N_{RAW}$, Slot Format and the Slot Duration Count *C* are also specified in the RPS element. The duration of the RAW slot $T_{slot}$ is defined by [2]:

(1) $$T_{slot}=500\;\mu s+C\cdot 120\;\mu s.$$

The Slot Duration Count *C* sub-field is either y=11 or y=8 bits long depending on whether the Slot Format sub-field is set to 1 or 0, respectively. The minimal RAW slot duration is thus 500 μs for C=0, whereas the maximal slot duration $T_{slot}^{max}$ is 246.14 ms for y=11 ($C=2^{11}-1$) and 31.1 ms for y=8 ($C=2^{8}-1$). The Number of Slots $N_{RAW}$ field is 14−y bits long. Consequently, the maximal number of slots $N_{RAW}^{max}$ is $2^{14-11}=8$ for y=11 and $2^{14-8}=64$ for y=8. Therefore, the maximal RAW group duration is $N_{RAW}^{max}\cdot T_{slot}^{max}$ and amounts to 1.9904 s for y=8 and 1.96912 s for y=11. It is important to note that (1) the RAW group duration must be shorter than the (short) beacon interval so RAW must be configured with respect to the (short) beacon interval and (2) if Cross-Slot Boundary (CSB) is forbidden (equals zero), the RAW slot duration must be at least as long as the packet transmission time, thus RAW must be configured with respect to the traffic patterns and distances in the network (i.e., packet sizes and MCSs). Otherwise, disrespecting (1) would cause the next beacon to cut-off the latter part of RAW (that continues after TBTT or TSBTT on the timeline), which would disable all the stations assigned to the cut-off slots to ever access the medium. Moreover, disrespecting (2) would disable every transmission or reception. If CSB is allowed (i.e., equals 1), a station is allowed to cross its assigned RAW slot boundary to complete the ongoing frame exchange sequence. Otherwise, a station will not transmit if the transmission would exceed the boundary of its allocated RAW slot. Stations determine the index of their assigned RAW slots as follows [2]:

(2) $$i_{slot}=\left(x+N_{offset}\;\mathrm{mod}\;N_{RAW}\right),$$

where $i_{slot}$ is the index of the RAW slot to which the station is mapped, $N_{RAW}$ is the total number of RAW slots in the corresponding RAW group, $N_{offset}$ is the offset value in the mapping function to improve fairness among the stations in a RAW, and *x* is the position index of the AID of the station if the RAW is restricted to stations whose AID bits in the TIM element are equal to 1, otherwise it is the AID itself.

**Figure 4:**
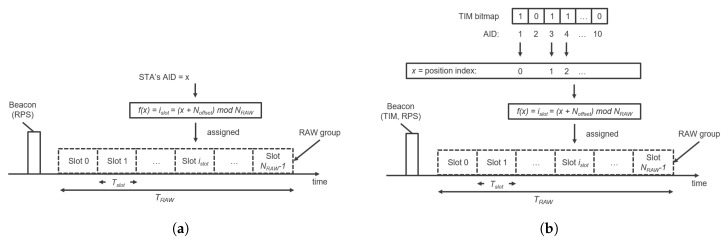
RAW slot assignment procedure when (**a**) RAW is not restricted to paged stations; (**b**) RAW is restricted to paged stations.

When the RAW is not restricted to stations with DL indication in the TIM element (paged stations), all stations that belong to a RAW group are allowed to access the medium in that RAW. Otherwise, only paged stations are allowed to access the medium in the RAW. After receiving a TIM element, the paged station starts to contend for the medium not earlier than its allocated RAW slot defined as the function of the station’s position in the TIM element and the RAW group information in the RPS element, whereas non-paged stations are not allowed to access the RAW. The RAW slot assignment procedure for both paged and non-paged stations is illustrated in Figure 4.

The RAW feature also introduced two back-off states for Enhanced Distributed Channel Access (EDCA) to manage transmissions inside and outside their assigned RAW slot, respectively. Lastly, RAW has two more operation modes: operation with Resource Allocation (RA) and Periodic RAW (PRAW) operation, which are beyond the scope of this paper. For more information, we refer the readers to [2].

## 4. Performance Evaluation and Discussion

To evaluate TIM and RAW performance with UL/DL traffic, we analyzed two scenarios: the first considers reliable and high-throughput traffic of IP camera streaming applications (cf., Section 4.2), whereas the second assesses the scalability of a reliable sensor network that sends measurements at specific time intervals (cf., Section 4.3). Exact simulation setups, obtained results, and their interpretations are presented in the corresponding sections. The ratio between uplink and downlink traffic amounts to 92/8% in the first scenario and 67/33% in the second scenario (in bytes). The general simulation setup is presented in Section 4.1, whereas Section 4.4 presents the discussion of the theoretical limits regarding throughput and latency in the simulated network (cf., Section 4.4). Finally, Section 4.5 discusses TCP behavior when the link layers introduce long delays.

### 4.1. Simulation Environment and Setup

We simulated the network in the ns-3 event-based network simulator that exhibits realistic propagation behavior (e.g., channel errors, capture effect). We used a standard log propagation loss model with values for outdoors scenarios and macro deployment [34]. We extended the IEEE 802.11ah module implementation [4] in ns-3 version 3.23 with a DTIM implementation, non-CSB operation and downlink frame scheduling at the AP. We updated the module from version 3.23 to version 3.25 and introduced an immediate reply mechanism for reducing the latency of DL frames (responses) that can be transmitted within the same RAW slot as the UL frame that triggered the response. This section presents the realized extensions of the ns-3 module by Tian et al. [4] and improvements in DL scheduling for bidirectional communication.

In the simulator, we implemented a two-level AID hierarchy. For example, if 100 stations are divided over four TIM groups, stations having AIDs 1–25 will belong to TIM group 0, stations having AIDs between 26–50 will belong to TIM group 1 and so on. Both the AP and stations only have one antenna. All simulations are performed with a beacon interval of 102.4 ms and a single RAW per beacon interval, where the RAW duration is maximized within that interval by maximizing the slot duration with respect to the configured number of RAW slots $N_{RAW}$. The same RAW configuration is used for all beacon intervals. The maximal slot duration is determined by the maximal possible RAW Slot Count and calculated according to the formula:

(3) $$C_{max}=\left\{\begin{array}{cc} q, & \mathrm{if}\;\left(q<256\wedge 8<N_{RAW}\leq 64\right)\vee \left(q<2048\wedge N_{RAW}\leq 8\right),\;\\ 255, & \mathrm{if}\;q\geq 256\wedge 8<N_{RAW}\leq 64,\\ 2047, & \mathrm{if}\;q\geq 2048\wedge N_{RAW}\leq 8, \end{array}\right.$$

where

(4) $$q=⎣\frac{\frac{T_{TIM}}{N_{RAW}}-500\;\mu s}{120\;\mu s}⎦,$$

and $0<N_{RAW}\leq 64$ ($T_{TIM}$ is expressed in μs). Note that, for very long beacon intervals, a single RAW group would not be able to cover the whole beacon interval due to constraints on the maximum number of slots and slot duration. However, multiple RAWs in one RPS could be used if we want RAWs to cover the entire beacon interval. All stations in one TIM group are assigned to the RAW within their corresponding beacon interval and distributed over the RAW slots. Thirty-two TIM groups are supported since we fixed bit-number $n_{1}$ in the AID field to 5. As such, the DTIM beacon contains a partial virtual bitmap indicating which TIM groups have DL data pending at the AP.

#### 4.1.1. DTIM Beacon

We implemented the DTIM beacon by sending the bitmap that indicates which TIM groups have DL data pending at the AP. Stations calculate the duration of the sleep time of their radio upon reception of the beacon. In reality, switching the radio from sleep to active mode takes time so we reduced the duration of the sleep time by 4 ms in order to provide the radio with enough time to switch from an off state to on and actively listening state. Furthermore, 4 ms is large enough to provide enough time even for radios with very long switch-on times, in the worst case. The time needed for switching from off to on state is configurable in the simulator to enable simulation of different radios.

To assess the energy consumption, we measured sleeping time and awake time of each station. Stations know if they can sleep through the entire TIM beacon period by checking the bitmap in the DTIM beacon and their own pending transmission queues. The radio is turned off at the physical layer and cannot sense any signal when stations enter sleep mode. Stations only wake up for the DTIM beacon, their TIM group beacon if their TIM group is paged in DTIM and their assigned slot in the RAW period (if they are paged in TIM or have data to transmit). This allows us to accurately assess the total sleep duration of the stations and prevent frames from being processed when they should not be.

#### 4.1.2. Downlink Frame Scheduling

There are two options for downlink data scheduling in IEEE 802.11ah. The first one is based on stations transmitting PS-Polls to retrieve downlink data, whereas the second one implies additional intelligence at the AP to calculate wake-up times for stations and to schedule downlink packets appropriately. We opted to implement the latter, namely transmission scheduling of downlink frames from the AP to the stations. DL packets must be sent during the corresponding RAW slot when the station is actively listening, otherwise the station will never receive them. When the AP MAC layer receives a packet for transmission from the layers above, it will check the destination of the packet and determine its corresponding AID. Based on the AID, the exact RAW slot can be determined and the AP will schedule the transmission at the beginning of the slot after the next DTIM beacon. This gives the AP the ability to indicate whether there is pending data for the RAW group of that destination and thus ensures the destination will be listening. To prevent the AP from sending data to stations outside their slots (for example due to backing off in heavy channel contention), we assigned a separate DCF manager with its own queue to each RAW slot. Each DCF manager will only have access to transmit its frames in the queue during the corresponding RAW slot, in exactly the same way the DCF managers of stations are restricted.

#### 4.1.3. Immediate Reply

We also improved bidirectional scheduling in IEEE 802.11ah by enabling the scheduling of immediate replies at the AP. If a station wakes up to transmit data in its corresponding RAW slot, it remains awake after the completion of the transmission until the end of the RAW slot. This enables the AP to check if the resulting reply can still fit in the same RAW slot. If so, the AP can immediately transmit the frame, and thus significantly reduce the round trip time. In case the reply does not fit in the current RAW slot, the AP is forced to schedule its transmission after the next DTIM beacon, prolonging the RTT for the length of the DTIM beacon interval. To be able to send immediate replies, the AP must keep track of whether stations are active by checking the last sent DTIM bitmap or whether data was received from the station during the current RAW slot.

In reality, it is unlikely for an AP to host the TCP server itself, introducing extra delay because of the RTT between the AP and the TCP server. For sufficiently large delays, the ability to reply within the same slot becomes infeasible. However, if the AP spoofs ACKs like in satellite networks [35] this problem could be somewhat mitigated. On the other hand, this could introduce other problems such as losses in the rare cases in which the spoofed ACK does not correspond to reality, i.e., in case that ACK was never received, or in case the re-transmission time is exceeded. In these cases, the spoofed ACK does not correspond with the actual fact that the packet never arrived.

#### 4.1.4. Non-Cross-Slot Boundary

Transmissions at the end of the RAW slot pose problems for bidirectional communication when non-CSB is used. As the transmission would go beyond the RAW slot, it would become impossible to receive the IEEE 802.11 ACK frame from the AP. Consequently, the device has to re-transmit anyway the next time channel access is granted. To prevent such unnecessary re-transmissions, we pass the RAW slot duration information to the DCA queue so that it can determine, based on the calculated transmission time and remaining RAW slot duration, whether or not a transmission would cross the boundary of the RAW slot. If the calculated transmission time is larger than the remaining RAW slot duration, the station will not contend for the medium and defer the transmission.

### 4.2. Video Streaming Scenario

Achieving reliability and high throughput is a challenge in IoT networks, especially over wider areas. Such networks could enable firmware updates over the air or high-throughput applications like video streaming. We evaluated the feasibility of reliable and high throughput traffic over IEEE 802.11ah by considering a video streaming scenario. In this scenario, the stations represent IP motion cameras with configurable parameters, including motion probability, duration to record if motion was detected and the data rate of the stream to be transmitted. This subsection presents attainable data rates of IP cameras on top of IEEE 802.11ah and the scalability of such a network (cf., Section 4.2.2) along with a description of the simulation setup (cf., Section 4.2.1).

#### 4.2.1. Video Streaming Scenario: Simulation Setup

In order to evaluate the limits on throughput and scalability in our video streaming network, we ensured there is always motion, so the cameras are streaming continuously. We varied six configuration parameters, as shown in Table 3, so as to analyze which parameters affect the attainable throughput most. We executed 19,008 ns-3 simulations to assess all combinations of the different parameters. Each simulation was 1000 s long.

*MCS* and bandwidth dictate the data rates and the attainable range. MCS0 with a 2 MHz channel has a theoretical limit of 650 kbps, MCS4 bumps it up to 3.9 Mbps and MCS8 is the fastest at 7.8 Mbps but is also the most constrained in terms of possible range (up to 100 m in outdoor environments).

*Contention per RAW slot* $c_{s}$ represents the number of stations with which station *x* shares its RAW slot. Contention introduces collisions and back-offs in shared RAW slots. A value of 0 means a station *x* has the channel to itself and does not need to contend. A value of 1 means a station shares its slot with 1 other station, and so on. This parameter allows us to generate a constant traffic demand regardless of the amount of TIM groups and RAW slots. As the stations are evenly distributed over TIM groups and RAW slots in RAWs, the total number of stations in the network is:

(5) $$N_{STAs}=\left(c_{s}+1\right)\cdot N_{TIM}\cdot N_{RAW}.$$

The AP schedules to next slot parameter enables forcing the AP to schedule each reply in the next available RAW slot (after the next DTIM beacon) instead of the current active one. This action introduces a delay that depicts realistic behaviour in case the TCP server is not running on the AP itself.

TCP segment size changes the payload size on the wire. Using a larger segment size increases the number of sent bytes before a TCP ACK from the server is expected, but also increases the number of collisions.

The parameters $N_{TIM}$ and $N_{RAW}$ stand for the number of TIM groups and the number of RAW slots, respectively. Varying $N_{RAW}$ will make the channel access time for stations smaller or larger because the duration of RAWs in each beacon interval is maximized to maximize channel access opportunities. In this constellation, with one TIM group (beacon interval is $T_{DTIM}=T_{TIM}=102.4\;$ ms) and five RAW slots, each station would have 20,420 μs of channel access each DTIM cycle, 51,140 μs for two slots and almost the full beacon interval with 102,380 μs for a single slot.

To measure the maximum attainable data rate along with maximal scalability, we measured both the sending rate at the station and receiving rate at the AP. When the receiving rate is lower than the sending rate, the EDCA queue and the TCP TX buffer fill up and packets start getting dropped, which is an indication of a congested network. The results of our simulations are presented and discussed in the following subsection.

#### 4.2.2. Video Streaming Scenario: Results

**Figure 5:**
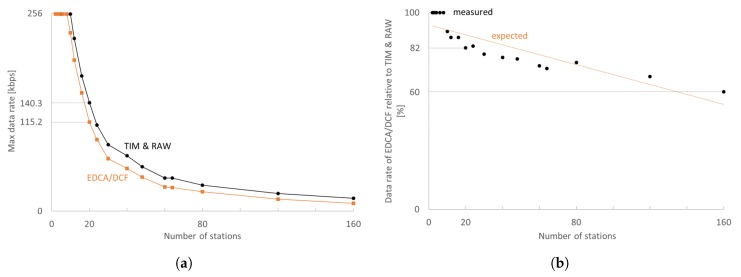
IEEE 802.11ah attains higher data rates per station with TIM segmentation and RAW slotting, and offers better scalability. (**a**) Maximum attainable data rates with TCP segment size 3216 bytes for MCS8 at 2 MHz over 200 m are higher with TIM segmentation and RAW slotting; (**b**) Maximum attainable data rate per stations with EDCA/DCF declines with increasing density relative to IEEE 802.11ah with TIM and RAW.

The trade-off between the maximum attainable data rate per station and the total number of cameras in the network is illustrated in Figure 5. The EDCA/DCF experiments have been performed with the same number of stations and the same effective medium access time. Already for 10 stations, TIM and RAW can achieve a 10% higher data rate than EDCA/DCF. For 10–80 stations, RAW and TIM introduce an improvement of at least 10–30% in maximum attainable data rate, whereas, for denser networks, this improvement further grows. However, low-quality video streaming is only feasible for up to 20 stations as the maximum attainable data rates become too low in denser networks. An average of 160 kbps per station is feasible with 20 stations, whereas 10 stations achieve 255.2 kbps with IEEE 802.11ah, which is significantly better than 115.2 kbps and 230.9 kbps achievable with EDCA/DCF, respectively.

**Figure 6:**
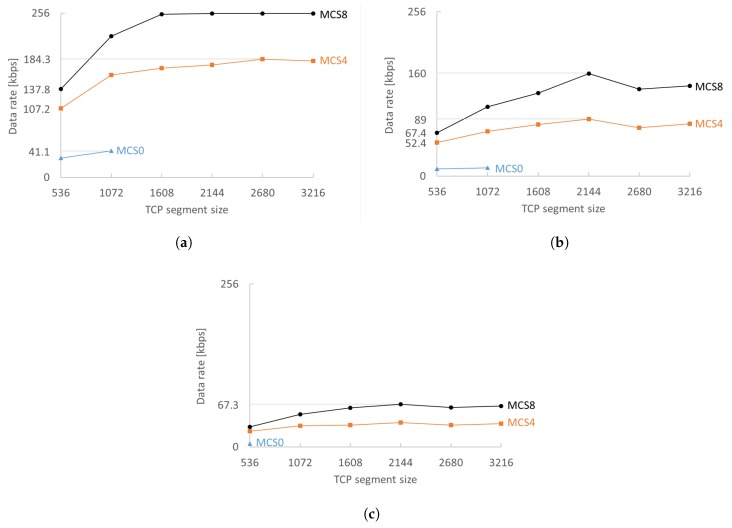
Average IP camera data rate per station for various TCP segment sizes and MCSs for (**a**) 10, (**b**) 20 and (**c**) 40 stations declines 45–70% when doubling the network density. Best overall performance is measured for a TCP segment size of 2144 bytes as it is the largest segment not fragmented at the network layer. (**a**) 10 stations; (**b**) 20 stations; (**c**) 40 stations.

The MCS and TCP segment size are, as expected, crucial for sustaining higher data rates. A higher MCS enables much faster transmissions, reducing the drops due to the busy radio (already transmitting/receiving). Only two stations can have 200 kbps with MCS0. A bigger TCP segment size makes better use of the available channel bandwidth before waiting for TCP ACKs, which can be clearly seen in the rise in achievable data rate (Figure 6). Experiments showed that, in general, 2144 byte TCP segments perform best in any configuration (regardless of density and MCSs). The only measured exception was for MCS4 with 10 stations as shown in Figure 6a. A TCP segment size of 2144 bytes performs best because it is the largest TCP segment that does not need IP fragmentation (as evaluated in Section 4.4).

Transmission of a single 1608 byte segment with MCS0 takes 21.84 ms, which is more than one fifth of our beacon interval. This means that, if RAW is used, its slots must be sufficiently long. TCP segment sizes larger than 1072 are not feasible with MCS0 for RAWs with more than four slots because the transmission time of a single segment would be longer than the RAW slot duration. With a maximum data rate of 41.1 kbps per station for 10 stations and 17.7 kbps for 20 stations, MCS0 is only feasible for less demanding applications. MCS4 fares better and attains a maximum of 89 kbps for 20 stations. MCS8 has the least problems to attain the receiving rate: a TCP segment size of 1608 bytes or higher is enough to reach 128 kbps data rate for 20 stations without any issue.

**Figure 7:**
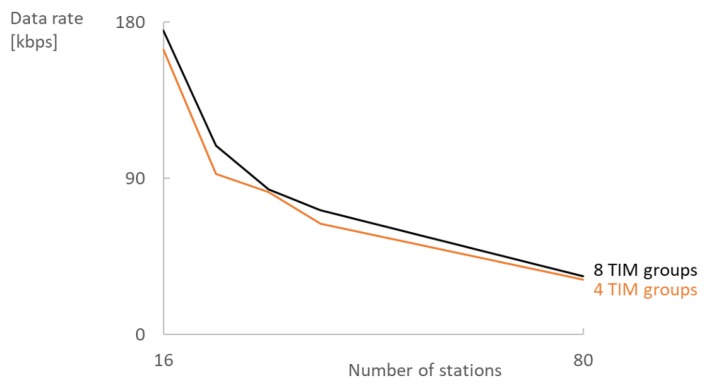
Increasing the number of TIM groups can increase the data rate 1–15% for MCS8.

Contention in RAW slots reduces the maximum attainable data rate by 45–70%, as can be seen by comparing graphs (a–c) in Figure 6. Stations sense if the medium is busy at the start of the RAW slot and, if so, apply the exponential back-off algorithm which on average halves the available time and thus the possible data rate. RAW standing alone largely contributes to reducing contention, but adding TIM segmentation makes the difference in dense (or high-throughput) networks where RAW meets its limits by further contributing to the reduction of contention. Increasing the number of TIM groups can increase the data rate by 1–15% for MCS8 (Figure 7) and 6–20% for MCS0. However, increasing the number of TIM groups has a serious impact on the latency: four TIM groups with a beacon interval of 102.4 ms result in a minimum RTT of at least 409.6 ms assuming the frame was queued in an empty EDCA queue during the RAW slot of the station and the AP schedules the reply in the next DTIM cycle. Doubling the number of TIM groups also doubles the round trip time that can pose problems for TCP retransmissions. If the streams have to be reliable but are generally unidirectional, then increasing the number of TIM groups is a better choice than increasing the contention inside a RAW slot. However, care must be taken when increasing the number of TIM groups because, at some point, latency will become too large and the network performance will degrade. The exact point depends on the network configuration and traffic load.

### 4.3. Reliable Monitoring Scenario

Closed-loop control over wireless is still a demanding challenge due to the stringent safety and security requirements in such systems. A step towards closing the loop in wireless IoT would be reliable and deliver sensor data on time to actuators. Therefore, we evaluated reliability and latency in a dense WSN scenario with up to 6960 stations connected to a single AP, where each transmitted packed is acknowledged at the application layer. Each node has a full TCP/IP stack implemented and connects to the server via TCP. This section presents the trade-off between attainable scalability and sampling rate per station. It also presents the influence of TIM and RAW on overall scalability and energy consumption in a scenario with one third of the traffic being downlink traffic. The main results are presented in Section 4.3.2, along with the simulation setup in Section 4.3.1.

#### 4.3.1. Reliable Monitoring: Simulation Setup

In this scenario, we analyze how many stations can be reliably supported by a single AP over a large area (up to 1 km) when using MCS0 and a 2 MHz channel. Each sensor station sends its measurements periodically to the TCP server at the AP. Each measurement consists of a payload of 100 bytes, corresponding to 190 bytes on the wire (26 B for WLAN quality of service data, 8 B for LLC, 20 B for IPv4 header, 32 B for TCP header, 100 B of data and four remaining bytes of the frame itself). Each measurement is acknowledged at the application layer (90 bytes). The payload size is also increased up to 400 bytes to evaluate the grouping multiple measurements together and sending them at a slower rate. The sampling interval $T_{s}$ is the time between two subsequent sensor measurement transmissions. Each station starts transmitting at a random moment within the sampling interval to prevent bursts of traffic and transmissions randomly deviate from the interval to simulate clock drift.

To determine the upper limit of attainable density, we gradually increased the contention per RAW slot by increasing the amount of channel traffic. The contention per RAW slot is defined as the number of stations with which station *x* shares its RAW slot (if there are *s* stations in a RAW slot, the contention is s−1). The attainable density is determined as the number of stations for which all stations can deliver their data reliably, without packet loss. If the amount of traffic is too large, i.e., in overly dense network, the EDCA queues will fill up and frames will be dropped. The contention per RAW slot is increased with increments of 5, so the maximum number of stations in the obtained results might be somewhat smaller than the real maximum, depending on the TIM and RAW configuration. The minimal contention that has been chosen is the value where all TCP connections remain established and the EDCA queues do not have more than one frame at the end of the simulation. The total number of stations can be calculated according to Equation (5). We assigned $N_{STAs}/N_{TIM}$ stations to the first TIM group only to reduce the simulation time. This action is motivated by the fact that each TIM interval contains the same RAW, so all TIM groups would behave equivalently. A 536-byte TCP segment size is used in this scenario because of the low data rate of MCS0.

We ran over 20,000 simulations with different configuration parameters listed in Table 4. Thirty servers with 2× Hexacore Intel E5645 (2.4 GHz) CPU (Virtual Wall testbed in Ghent, Belgium), 24 GB RAM, 1 × 250 GB hard disk and 1–5 gigabit nics per node were used for this endeavor, as each ns-3 simulation with very large number of stations can take several hours to complete.

#### 4.3.2. Reliable Monitoring: Results

**Figure 8:**
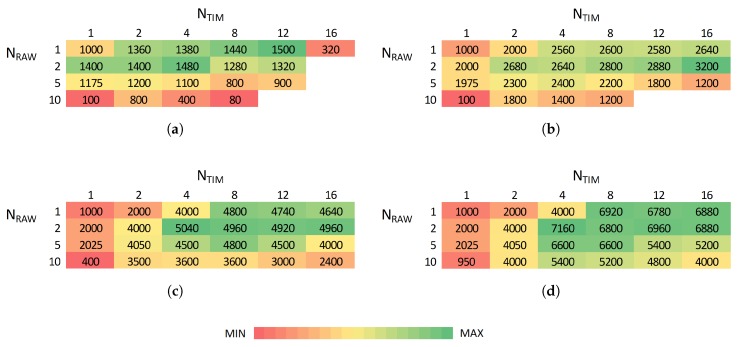
The highest density without any packet loss is achieved with high number of TIM groups and low number of RAW slots, depending on sampling rate (traffic load). (**a**) $T_{s}=10$ s; (**b**) $T_{s}=20$ s; (**c**) $T_{s}=40$ s; (**d**) $T_{s}=60$ s.

Figure 8 shows, for different sensor sampling rates, the maximum number of stations that can be supported for different combinations of the number of TIM groups and RAW slots. Intuitively, achievable density generally does not decrease when decreasing sampling rates (decreasing overall traffic load) as shown in Figure 8. For the smallest evaluated sampling interval (10 s), the use of 16 TIM groups already introduces too much latency, leading to a rapidly decreasing maximum attainable density. However, the highest density is generally achieved for many TIM groups and a few RAW slots. At least 1500, 3200, 3800, 4960, 6120 and 6960 stations can reliably report every 10, 20, 30, 40, 50 and 60 s, respectively, with no packet loss.

**Figure 9:**
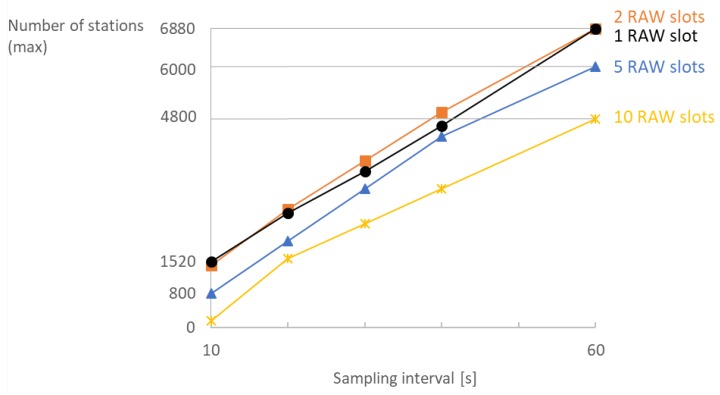
Extensive RAW slotting limits the maximum attainable density in a network with 16 TIM groups.

Consequently, there is a trade-off between achievable density and traffic load. IEEE 802.11ah can support thousands of stations as long as each of them has limited traffic demands. In that manner, the relation between attainable density and sampling interval is almost linear as shown in Figure 9. Increasing the number of RAW slots limits the available channel time and thus limits the scalability. RAW slotting is beneficial for reducing contention; however, it can be limiting if bidirectional traffic is present as it postpones transmissions for paged stations. Hence, reliable bidirectional communication among 6880 stations with a sampling interval of 60 s and 16 TIM groups is possible for 1 and 2 RAW slots, whereas for 10 slots it is only possible only among 4800 stations.

**Figure 10:**
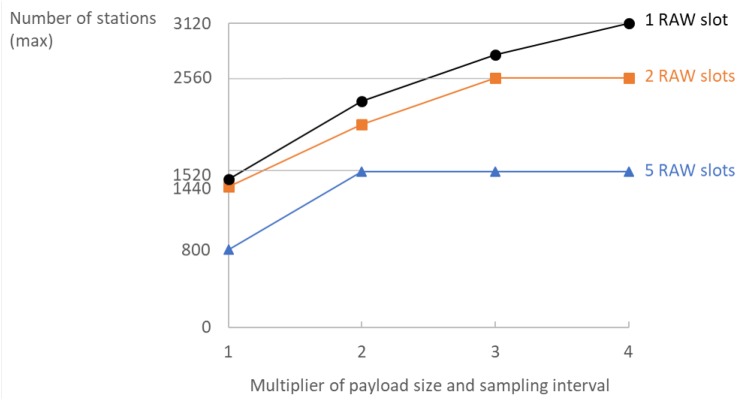
Grouping *n* measurements and sending them *n* times less frequently benefits the maximum attainable density in a network with 16 TIM groups.

Multiplying the sampling interval and payload size by the same factor is beneficial for the overall scalability. While the overall reception rate at the server will remain the same, the maximum density rises considerably by grouping *n* measurements together and reporting them less frequently (every n·10 s) as shown in Figure 10. A larger packet size makes better use of the available channel time: it reduces the overhead of the headers, the amount of TCP acknowledgements and the number of collisions. For five RAW slots, grouping two measurements together and reporting them twice less frequently enables support for twice as many stations. For two RAW slots, this difference is somewhat smaller but grouping up to 3100-byte measurements together and reporting them up to three times less frequently enables an additional 1120 stations. We tested grouping up to four measurements, since any larger values would result in exceeding the 536 bytes TCP segment size and would cause more traffic due to IP fragmentation. On the other hand, increasing the packet size makes the use of configurations with a large number of short slots impossible, due to infeasibility to transmit (receive) large packets in too short slots as discussed in Section 4.4. The possibility of grouping measurements together and reporting them less frequently greatly depends on real-world application. In delay-intolerant systems, this might not be possible; however, it is greatly beneficial for scalability when possible.

**Figure 11:**
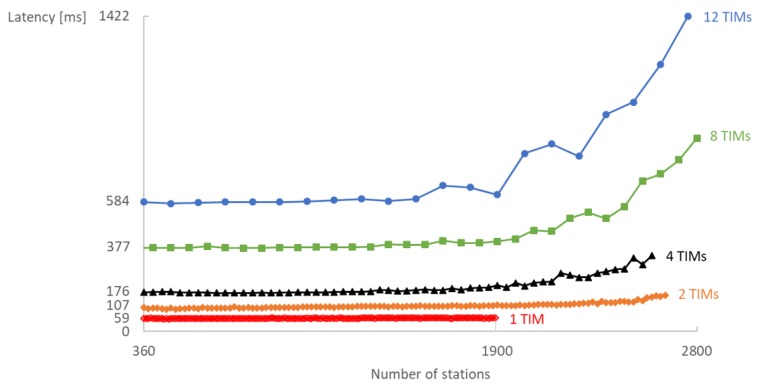
Increasing the number of TIM groups considerably increases the average latency in a network with a fixed number of RAW slots ($N_{RAW}=2$) and sampling interval ($T_{s}=20$ s). The average latency diverges when the traffic load becomes too high.

Increasing the number of TIM groups instead improves scalability but also increases latency considerably (Figure 11) and RTT even more. The average latency starts to diverge when the traffic load becomes too high. For $T_{s}=20$ s and 12 TIM groups, the average latency rapidly grows already from 1920 stations as illustrated in Figure 11. In addition, TCP should be tuned, i.e., the TCP timeout should be increased, to properly handle the longer delays caused by increasing the number of TIM groups.

**Figure 12:**
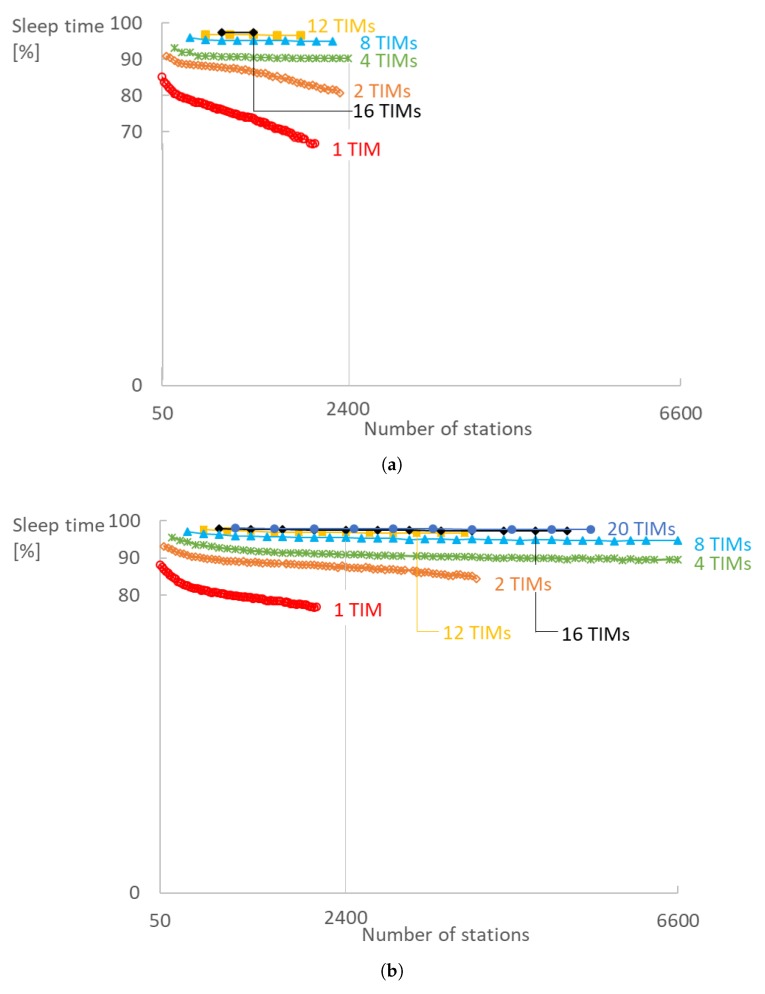
Increasing the number of TIM groups enables stations to sleep longer. Stations sleep over 90% of the time already from four TIM groups and above, with five RAW slots and both (**a**) 20 s and (**b**) 60 s sampling interval. (**a**) $T_{s}=20$ s; (**b**) $T_{s}=60$ s.

TIM and RAW configurations also have a big impact on the sleeping time of the radio. We evaluated the energy consumption by measuring the total time the radio is turned off at the physical layer (Figure 12). Our measurements do not take into account the interference of other stations (collisions) since in our model a station is awake for the whole duration of its corresponding RAW slot if it does not go to sleep immediately after the beacon. A station goes to sleep immediately after the DTIM beacon if it neither has data to transmit nor is paged. Else, it also wakes up to receive the TIM beacon. A station goes to sleep immediately after the TIM beacon if it is not paged and has no data to transmit. Generally, the higher the contention within a RAW slot, the more active each radio has to be. Worst case, the radio has to be active for a RAW slot duration each DTIM cycle, for each DTIM beacon and for each TIM group beacon the station belongs to. The sleep time decreases more rapidly as soon as RAW slots become fully saturated and transmissions need to be rescheduled to slots in the next DTIM cycle as is evident from Figure 12 for 1 and 2 TIM groups. In addition, 97% of time station spend sleeping in a configuration with 16 TIM groups, five RAW slots and sampling interval of 10 s.

**Figure 13:**
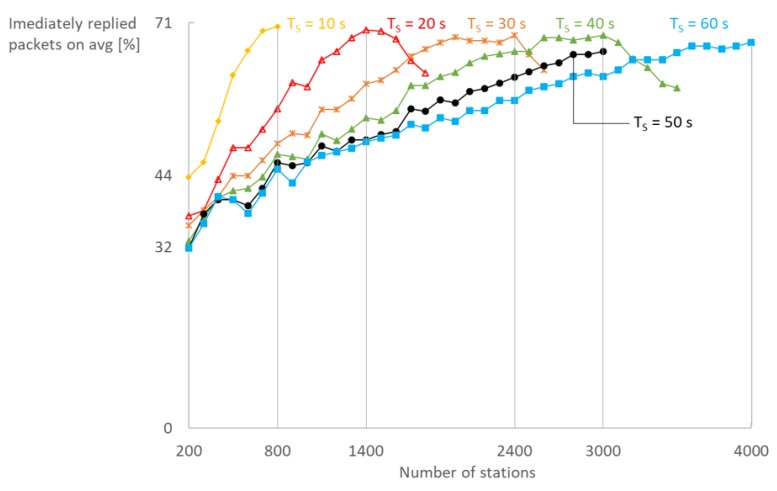
With two TIM groups and 10 RAW slots, up to 70% of all downlink packets can be immediately transmitted (AP reply) avoiding the need to wait until after the next DTIM beacon, thus halving the RTT.

Finally, the use of our immediate reply mechanism in bidirectional communication greatly reduces RTT and is scalable as shown in Figure 13. The average number of immediate replies per station grows until saturation of the network is reached, after which it decreases with the same rate. Up to 71% of the replies can be scheduled in the same RAW slot as the request which at least halves the RTT, having in mind that the reply would otherwise be scheduled in the next DTIM cycle.

### 4.4. Theoretical Limits

The duration of a single packet transmission $t_{TX}$ at the 2 MHz channel with a short preamble is:

(6) $$t_{TX}=240\;\mu s+40\;\mu s\cdot N_{SYM},$$

with 240 μs being the sum of preamble duration (160 μs) and the SIGNAL field duration (80 μs), 40 μs the Orthogonal Frequency-Division Multiplexing (OFDM) symbol duration with normal guard interval and $N_{SYM}$ the number of symbols for Binary Convolutional Code (BCC) encoding defined as:

(7) $$N_{SYM}=⎡\frac{22bits+8\cdot l}{N_{DBPS}}⎤,$$

where

(8) $$N_{DBPS}=DR\cdot 40\;\mu s.$$

In Equations (7) and (8), 22 bits is the sum of bit-lengths of the *Service* field and the tail bits per BCC encoder, *l* is the packet-size in bytes and DR is the data rate. The time needed to ensure successful transmission $t_{TX}^{total}$ includes SIFS (160 μs) and ACK (480 μs) durations, and is expressed as:

(9) $$t_{TX}^{total}=t_{TX}+160\;\mu s+480\;\mu s.$$

The RAW slot duration must be longer or equal to the transmission time of a segment (cf., Table 5) in order to allow a non-zero probability for successful transmission. The maximum number of transmissions in one beacon interval is:

(10) $$N_{{TX}_{max}}^{b}=⎣\frac{N_{TIM}\cdot T_{TIM}}{t_{TX}^{total}}⎦.$$

Assuming all packets have the same size on average and RAW(s) is (are) occupying the entire channel time.

The packet-size *l* in the IP camera experiments equals the sum of the payload encapsulated in the TCP segment and 90 bytes of overhead (32 bytes for the TCP header, 20 bytes for the IPv4 header, eight bytes for LLC, 26 bytes for WLAN quality of service data and four bytes for the frame) if there is no IP fragmentation. 2680 and 3216 byte TCP segments need IP fragmentation and are broken into two smaller data segments: 2272+440 for 2680-byte segments and 2272+976 for 3216-byte segments. This fragmentation results in an overhead of 58 bytes per fragment (32 bytes of TCP header are included in fragments). The transmission duration for each fragment is then calculated using the packet size of the fragment (i.e., 2272 bytes of data +58 bytes of overhead). Note that $N_{{TX}_{max}}^{b}$ successful transmissions cannot be guaranteed only with allowing enough time in RAW because stations employ random channel access. Khorov et al. [27] proposed a model for calculating of transmission success probability. However, they assume each station has only one packet to transmit during its RAW slot (only uplink traffic, without TIM); thus, the model is not applicable for the case with TIM segmentation and DL data.

The round trip time is largely influenced by TIM segmentation. In our experiments, stations exchange data with a server at the AP. Therefore, the RTT is at least $2T_{DTIM}$, unless an immediate reply is implemented. If stations would partake in bidirectional communication with each other, the minimal RTT would be $4T_{DTIM}$ since four hops are needed for a packet to complete the loop. Due to random channel access, these times will be often longer. Therefore, $T_{DTIM}$ must be at least two or four times smaller than the smallest sampling interval in the sensor network in order to complete one transaction before the start of the next one, as in a reliable monitoring scenario.

Taking into account the previous calculations, the maximum possible data rates achievable in reality are somewhat smaller than those specified by the standard. The maximum possible data rate for MCS8 (specified data rate is 7800 kbps) and 32 TCP segments of 2144 bytes (resulting in full channel utilization) per cycle is thus 5505 kbps according to:

(11) $$DR_{p}^{max}=\frac{N_{{TX}_{max}}^{b}\cdot \left(l_{p}+90B\right)}{102.4\;\mathrm{ms}},$$

where $l_{p}$ denotes the payload size. However, since RAW restricts channel access time for each station, the maximal effective data rate per station is:

(12) $$DR_{eff}^{max}=\frac{DR_{p}^{max}\cdot T_{slot}}{N_{TIM}\cdot 102.4\;\mathrm{ms}}.$$

Thus, for 5-slot RAW, four TIM groups and a 2144 bytes TCP segment, the maximum effective data rate per station is 274.44 kbps. Due to stochastic channel access and downlink data scheduling, the experimentally achieved data rate is 41.7% lower (160 kbps) in this scenario. Existence of DL data can largely reduce the available channel time for UL since for every RAW group with at least one paged station, and full RAW is used only for DL unless DL data is immediate AP response. Evidently, network density, TCP, TIM and RAW configurations must be appropriately chosen in order to achieve a targeted throughput.

### 4.5. Delay-Tolerant TCP

TCP has been designed to avoid network congestion and treats all losses as such, irrespective of the cause being network propagation delay or losses due to collisions. Frequent time-outs can occur if TCP is not adjusted to the long propagation delays. Frequent time-outs would result in a build-up of (unnecessary) re-transmissions that such a network could not handle in an acceptable time, causing a significant reduction of the throughput. With the slow start mechanism, it would take a long time to get back to the optimal congestion window size. There are TCP implementations that can handle high latency networks, such as satellite networks, more gracefully such as TCP Peach [36] or TCP Noordwijk [37]. The delays introduced by waiting for the RAW slot during which the transfer can take place can become comparable to high latency networks, making these enhancements also useful to be deployed in IEEE 802.11ah environments. Unfortunately, ns-3 does not yet provide these implementations, so TCP Westwood [38] has been used instead, which is suitable for lossy wireless connections.

**Figure 14:**
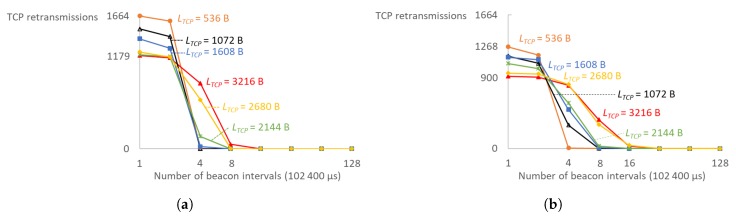
To avoid build-up of unnecessary TCP retransmissions due to long delays on link layer, minimum Retransmission Timeout (RTO) should be set high enough for each MCS as illustrated for (**a**) MCS8 and (**b**) MCS4. (**a**) The minimum RTO should be as long as eight beacon intervals when 3216- and 2680-byte TCP segments are used with MCS8. For shorter TCP segments, the RTO should not be smaller than four beacon intervals; (**b**) The minimum RTO should not be shorter than 16 beacon intervals when 3216- and 2680-byte TCP segments are used with MCS4. For shorter TCP segments, the RTO should not be smaller than eight beacon intervals, except for 536-byte TCP segments that allow four beacon intervals.

The minimum Retransmission Timeout (RTO) is set high enough such that TCP re-transmissions do not influence the results due to timeouts. Normally, the TCP congestion algorithm is smart enough to adjust the congestion window and RTO values based on the measured RTT to match the possible throughput. However, this would require a period where lots of re-transmissions are being sent until the parameters settle, which would skew the results. If TCP re-transmissions occur in an already saturated channel, it would create a snowball effect where more re-transmissions are sent until the maximum amount of re-transmission attempts have been exceeded and the TCP connection is ultimately dropped. The results should be interpreted as the absolute maximum limit. In these tests, the IEEE 802.11 ACK and subsequent re-transmission on a missed ACK check as part of in the MAC implementation was sufficient to recover lost frames, but, in real-life scenarios, a certain margin should be taken into account to allow for a graceful increase in channel traffic due to TCP re-transmissions. As shown in Figure 14, a minimal RTO value of a single beacon interval is vastly insufficient. Larger TCP segments need larger minimal RTOs in order to bring re-transmissions down to a minimum: eight beacon intervals (819.2 ms) for MCS8 at 2 MHz channel and 16 (1.6384 s) for MCS4 at 2 MHz. For smaller TCP segments, four beacon intervals (409.6 ms) are small enough for minimal RTO. Almost all re-transmissions were eliminated after a minimal RTO value of 1.6384 s or four DTIM cycles with four TIM groups, and only MCS0 required twice larger value.

## 5. Conclusions

This article presents the influence of the joint usage and performance of TIM and RAW on scalability, throughput, latency and energy efficiency in networks with bidirectional traffic. In the presence of downlink traffic, the RAW mechanism limits uplink performance by rescheduling it to the next available channel access opportunity and giving precedence to downlink. The RAW performance in uplink-only networks is different than in the ones with downlink or bidirectional traffic. We conducted over 40,000 experiments in the ns-3 simulator to evaluate the behaviour of TIM and RAW in the presence of bidirectional traffic.

We envisioned this IoT technology as a long-range technology that is able to support legacy Internet traffic and therefore evaluated the full network stack. IEEE 802.11ah requires more changes at the various layers of the network stack than other 802.11 standards. The network and transport layer requires adjusted settings to incorporate the introduced delays due to intermittent channel access and the application layer has to limit the data transfer to prevent buffer overflows. However, when implemented properly, the promised power saving, range extension and higher station limit work very well without crippling the use cases it has been designed for. TCP adds extra header overhead, but it is still feasible to support legacy Internet traffic in IoT. IEEE 802.11ah offers a good balance between throughput and distance in comparison with LPWAN and WPAN technologies. It enables reliable communication since there are no exclusive constraints on downlink traffic.

TIM segmentation contributes to both high scalability and energy preservation and, combined with the RAW mechanism, they can enable reliable and high-throughput applications such as IP camera streaming and over-the-air firmware updates for up to 20 stations over 200 m with 160–256 kbps of throughput per station. TIM segmentation and RAW dominantly define the network performance. However, the standard does not specify how to configure them. The performance of both TIM and RAW are greatly dependent on traffic patterns and improper configuration could degrade the overall network performance. Proper TIM and RAW configurations introduce an approximately linear improvement in throughput with density in comparison with EDCA/DCF and represented by:

(13) $$I_{\%}=0.2529\cdot N_{STAs}+6.2074,$$

where $I_{\%}$ is throughput improvement in percentages introduced by TIM and RAW and $N_{STAs}$ is total number of stations. By enlarging the TCP segment size to a maximal value that is not subject to IP fragmentation, the maximal attainable throughput for a given MCS can be reached. The number of RAW slots defines the amount of contention in each slot, with respect to the number of stations. Ergo, RAW has the largest influence on the maximum attainable data rate in IEEE 802.11ah. However, in networks with high traffic loads or high density, combining TIM with RAW can further improve the data rates up to 15% if the DTIM interval is still small enough to not cause extensive TCP re-transmissions.

Increasing the number of TIM groups but limiting the number of RAW slots improves the overall scalability as found in the reliable monitoring scenario. In addition, 6960 stations reporting 100-byte measurements every minute is supported with 12 TIM groups and two RAW slots without packet loss. Extensive RAW slotting can be a limiting factor for bidirectional traffic since it postpones transmissions for paged stations. This can lead to quicker buffer overflow.

Grouping several measurements into one packet and sending them less frequently benefits the overall scalability. A larger packet size makes better use of the available channel time: it reduces the overhead of the headers, the amount of TCP acknowledgements and the collisions. With 10 s sampling interval and 100-bytes payload, 1440 stations can be supported with no packet loss, whereas grouping two or three such measurements together and sending them two or three times less frequently results in 2080 or 2560 stations, respectively.

IEEE 802.11ah enables all 5500 stations to turn off the radio for 97% of the total simulation time when using 20 TIM groups, five RAW slots and sampling interval of 60 s. With 10 s, 1800 stations can also sleep 97% of the time using 12 TIM groups and 5 RAW slots. Already from four TIM groups, sleeping time is over 90% and almost constant. However, doubling the number of TIM groups increases latency almost twice and doubles the RTT. There is a trade-off between energy efficiency and latency.

Considering the presented evaluation, the best approach to maximize the efficiency of a bidirectional communication would be:

1. Choose a sampling interval that is as high as possible, but still acceptable for the application. Furthermore, if it is acceptable to delay transmissions by grouping multiple measurements together into a bigger packet, it is advised to do so. If TCP is used, group measurements as such to fill the largest non-fragmented TCP segment size.
2. Choose the number of TIM groups so that $T_{DTIM}$ is at least four times smaller than the smallest sampling interval in the network.
3. In the chosen interval and TIM group, maximize the number of RAW slots so that they are long enough to be able to serve stations assigned to them, having in mind the maximal possible number of transmissions in a single slot.

Our immediate reply mechanism greatly improves bidirectional communication. In the presence of downlink traffic, RAW limits uplink by rescheduling it to the next available channel access opportunity and giving precedence to downlink. Our mechanism not only halves the RTT of 30–70% of packets by scheduling the reply from the AP in the current RAW slot if possible, but also enables uplink packets to be transmitted in the next cycle by eliminating paged stations and allowing stations to attempt uplink transmission (stations are not paged in the next cycle because they already got DL packet). Higher improvements are attainable in non-saturated networks, whereas, in highly dense networks with an almost fully utilized channel, there is no time left in RAW slots to schedule replies, decreasing the potential improvement.

**Figure 15:**
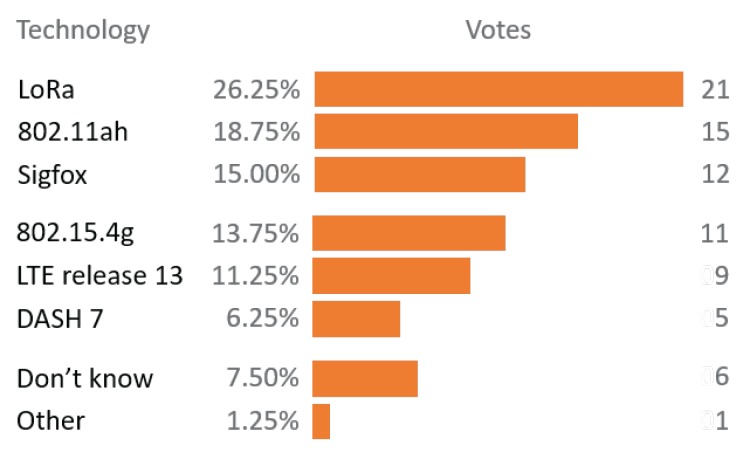
In 2016, IEEE 802.11ah was voted the second most interesting technology by Industrial Advisory Committee (IAC) members in spite of the lack of hardware support at the time.

Finally, this evaluation and obtained results enable smart usage of TIM segmentation and the RAW mechanism in reliable and high-throughput applications, as well as in reliable low-throughput applications with bidirectional communication. We aim to use this evaluation to design a self-adaptable network management algorithm to enable rea-time TIM and RAW regrouping, which would optimize throughput and/or latency and/or energy efficiency in closed loop scenarios. Judging by the conducted survey among Industrial Advisory Committee (IAC) members (Figure 15), there will be much more research on this technology and we will get to see its grand rise as it was the only technology from the survey not available on the market at the time, and yet rated the second most interesting.

## Figures and Tables

**Table 1:** The majority of available IEEE 802.11ah MAC optimization algorithms optimize only RAW and consider only uplink traffic.

Reference	Year	Ttraffic	MAC Features	Objective	Validation Tool
*This article*	2018	UL/DL	TIM & RAW	reliability, latency, throughput & energy	ns-3
Kureev et al. [11]	2017	UL/DL	TIM & RAW	energy & throughput	analytical & unknown simulation
Kim and Chang [12]	2017	UL/DL	TIM & RAW	energy	Matlab
Bankov et al. [13]	2016	UL/DL	TIM & RAW	energy & throughput	analytical & unknown simulation
Bel et al. [14]	2014	UL/DL	TIM & RAW	energy	Matlab
Badihi et al. [15]	2016	DL	TIM	latency	unknown simulation
Damayanti et al. [16]	2016	DL	TIM & RAW	hidden node mitigation	Matlab
Charania et al. [17]	2017	UL	TIM & RAW	latency and energy	Matlab
Nawaz et al. [18]	2017	UL	RAW	throughput	analytical
Tian et al. [19,20]	2017	UL	RAW	throughput	ns-3
Beltramelli et al. [21]	2017	UL	RAW & TWT	latency and energy	analytical
Wang et al. [22]	2017	UL	RAW	energy	Matlab
Yoon et al. [23]	2016	UL	RAW	hidden node mitigation	analytical & unknown simulation
Dong et al. [24]	2016	UL	RAW	hidden node mitigation	Matlab
Chang et al. [25]	2015	UL	RAW	throughput	unknown simulation
Wang et al. [22,26]	2015	UL	RAW	energy	Matlab
Khorov et al. [27]	2015	UL	RAW	throughput	analytical
Qutab-Ud-Din et al. [28]	2015	UL	RAW	energy and throughput	analytical
Park et al. [29]	2014	UL	RAW	throughput	analytical & unknown simulation
Raeesi et al. [30]	2014	UL	RAW	throughput and energy	analytical
Zheng et al. [31]	2014	UL	RAW	throughput	analytical & unknown simulation
Ogawa et al. [32]	2013	UL	unknown	throughput & energy	unknown

**Table 2:** IEEE 802.11ah has a number of novel MAC features in comparison with other Wi-Fi amendments [10].

Notable Features		802.11-2007	802.11n	802.11ac	802.11ah
Backwards compatibility		X	X	X	
Distributed Channel Access (DCF)		X			
Point Coordinated Function (PCF)		X			
Hybrid Coordination Function (HCF)	HCCA	X	X		X
	EDCA	X	X	X	X
Transmission Opportunity (TXOP)	Forward	X	X	X	X
	RD Protocol		X	X	X
	BDT				X
Response Indication Deferral (RID)					X
Frame Aggregation			X	X	X
Block Acknowledgement		X	X	X	X
Multi User (MU) Aggregation				X	X
Null Data Packet (NDP)			X	X	X
Group–ID				X	X
BSS color					X
Dynamic Bandwidth Management				X	
Subchannel Selective Transmission				X	X
Traffic Indication Map (TIM)		X	X	X	X
Delivery Traffic Indication Map (TIM)			X	X	X
Target Wakeup Time (TWT)					X
Grouping of Stations					X
Hierarchical AID					X
Dynamic AID reassignment					X
Restricted Access Window (RAW)					X
Group sectorization					X
Relay operations					X
Power saving at AP					X
Low power mode of operations					X

HCCA—HCF Controlled Channel Access; EDCA—Enhanced Distributed Channel Access; RD—Reverse Direction; BDT—Bidirectional TXOP.

**Table 3:** Varied configuration parameters in video streaming scenario.

Parameter	Values									
MCS (2 MHz)	MCS0	MCS4	MCS8							
Contention per RAW slot	0	1	2	3						
AP schedules to next slot	true	false								
IP camera sending rate (kbps)	2	4	8	16	32	48	64	96	128	256
TCP segment size	536	1072	1608	2144	2680	3216				
$N_{TIM}$	1	2	4	8						
$N_{RAW}$	1	2	5							

**Table 4:** Configuration parameters for the reliable monitoring scenario. High payload sizes (marked with *) have only been evaluated with 8, 12 and 16 TIM groups.

Parameter	Values					
Traffic interval (s)	10	20	30	40	60	
Contention per RAW slot	9	14	19	24	..	999
$N_{TIM}$	1	2	4	8	12	16
$N_{RAW}$	1	2	5	10		
Payload size (bytes)	100	200 *	300 *	400 *		

**Table 5:** Transmission durations $t_{TX}^{total}$ of various-sized TCP segments determine maximum possible number of segments to be transmitted in one beacon interval $N_{{TX}_{max}}^{b}$ ($N_{TIM}=1$, $T_{TIM}=102.4$ ms) for various MCSs and using a 2 MHz channel.

TCP Segment Size	536		1072		1608		2144	
**MCS**	$t_{\mathrm{TX}}^{\mathrm{total}}$ **(ms)**	$N_{{\mathrm{TX}}_{\max}}^{b}$	$t_{\mathrm{TX}}^{\mathrm{total}}$ **(ms)**	$N_{{\mathrm{TX}}_{\max}}^{b}$	$t_{\mathrm{TX}}^{\mathrm{total}}$ **(ms)**	$N_{{\mathrm{TX}}_{\max}}^{b}$	$t_{\mathrm{TX}}^{\mathrm{total}}$ **(ms)**	$N_{{\mathrm{TX}}_{\max}}^{b}$
MCS0	8.64	11	15.24	6	21.84	4	28.44	3
MCS1	4.76	21	8.08	12	11.36	9	14.68	6
MCS2	3.48	29	5.68	18	7.88	12	10.08	10
MCS3	2.84	36	4.48	22	6.12	16	7.8	13
MCS4	2.2	46	3.28	31	4.4	23	5.48	18
MCS5	1.88	54	2.68	38	3.52	29	4.36	23
MCS6	1.76	58	2.48	41	3.24	31	3.96	25
MCS7	1.68	60	2.32	44	3	34	3.64	28
MCS8	1.56	65	2.08	49	2.64	38	3.2	32
